# Autologous Adipose Tissue vs. Platelet-Rich Plasma for Treatment of Knee Osteoarthritis

**DOI:** 10.3390/biomedicines10102527

**Published:** 2022-10-09

**Authors:** Ashim Gupta

**Affiliations:** 1 Regenerative Orthopaedics, Noida 201301, UP, India; ashim6786@gmail.com; 2 Indian Stem Cell Study Group (ISCSG) Association, Lucknow 226010, UP, India; 3 Future Biologics, Lawrenceville, GA 30043, USA; 4 BioIntegrate, Lawrenceville, GA 30043, USA; 5 South Texas Orthopaedic Research Institute (STORI Inc.), Laredo, TX 78045, USA

Osteoarthritis (OA) is a tremendously widespread joint ailment, typically affecting large weight-bearing joints and influencing over 30 million individuals in the United States, with the anticipated number of patients to reach 67 million by 2030 [1]. Its pathophysiology is associated with synovial tissue inflammation and articular cartilage degeneration, causing pain and diminished function [1,2,3]. Generally, OA is handled with physical therapy, activity alteration, pharmacological agents (for example, non-steroidal anti-inflammatory drugs, opioids, corticosteroids, viscosupplementation, etc.), and surgery once established treatment approaches have failed [1]. These aforementioned treatment modalities have limits, incessantly seeking to lessen pain in place of targeting the underlying pathology [1].

Recently, numerous molecular targets, such as interleukin-1 (IL-1), transforming growth factor-β (TGF-β), matrix metalloproteinases (MMPs), etc., have been described as being implicated in the etiopathogenesis of OA [4,5,6]; still, various therapies may well have a negative risk-to-benefit ratio [7,8]. As a result, additional safe and effective treatment alternatives are needed to manage this unmet medical necessity.

Recently, there has been increased interest in the use of autologous biologics, including platelet-rich plasma (PRP), adipose tissue, etc., for regenerative medicine applications, especially in musculoskeletal conditions such as knee osteoarthritis [9]. Numerous randomized controlled trials and meta-analyses have shown the safety and efficacy of PRP for the treatment of knee OA [10]; thus, PRP is considered the gold-standard biologic for the treatment of musculoskeletal injuries. On the other hand, other autologous treatment modalities such as adipose tissue (including microfragmented adipose tissue (MFAT)) have not undergone a similar degree of clinical trials [10]. Initial clinical studies have reported improved patient-reported outcomes in patients suffering from knee OA post-administration of MFAT [11,12,13]. Moreover, a study from Dallo et al. reported that outcomes post-administration of MFAT were superior to three injections of PRP + viscosupplementation at 6-month follow up. However, this was one of the first prospective studies to compare the efficacy of MFAT with PRP, and various factors, including use of inferior-quality PRP, the addition of viscosupplementation, etc., complicated the conclusions from this study [14]. In this editorial, I focused on a recently published, one of the first, randomized controlled trials, titled “Platelet-Rich Plasma Versus Microfragmented Adipose Tissue for Knee Osteoarthritis: A Randomized Controlled Trial”, where the patient-reported outcomes of a single injection of PRP were compared to MFAT for knee OA [10].

In this study by Baria et al. (ClinicalTrials.gov identifier: NCT04351087) [10], a total of 58 patients with symptomatic knee OA (Kellgren–Lawrence (KL) scale grade 1–4) were randomized 1:1 to receive either a single injection of leukocyte-rich PRP or MFAT. PRP was made by processing 156 mL of the whole blood. MFAT was produced by aspirating 30 mL of adipose tissue via the traditional lipoaspiration technique. Patient-reported outcome measure (PROM) scores for the Knee injury and Osteoarthritis Outcome Score (KOOS) subscales (Pain, Symptoms, Activities of Daily Living, Sport and Recreation, and Quality of Life); visual analog scale for pain with Activities of Daily Living (VAS-ADL); and Tegner activity scale were documented at baseline and at 1, 3, and 6 months post-injection. Patients were also examined for adverse events, with an extra 2-week wound check for the MFAT group. The primary outcome was the KOOS-Pain subscore at 6 months post-injection. Cellular analysis (complete blood count) and growth factor determination was also performed for PRP via hemoanalyzer and ELISA assay, respectively. In addition, the number of total nucleated cells was also determined utilizing trypan blue dye and hemocytometer for the MFAT group. No significant differences between the groups were observed in terms of age, body mass index, or race at baseline. The PRP group (N = 30) had a mean volume of 5.12 ± 1.12 mL injected. This comprised a mean platelet count of 2673.72 ± 1139.04 × 10^3^/µL and a mean leukocyte count of 25.36 ± 13.27 × 10^3^/µL. The differential leukocyte count consisted of 67.81% lymphocytes, 18.66% monocytes, and 12.33% neutrophils. The MFAT group (N = 28) had a mean volume of 7.92 ± 3.87 mL injected, a mean total nucleated cell count of 3.56 ± 4.62 million/mL, and cell viability of 97.96%. In both groups, at the 6-months follow up, all of the KOOS subscales (including the primary outcome, KOOS-Pain) and VAS-ADL scores show significant improvement compared to baseline. However, no significant differences were observed between the groups in the final KOOS-Pain subscores or in the other PROM scores. This study was not without limitations, as also discussed by the authors of the study. These include insufficient power (small sample size) to detect differences between the groups, the short duration of the follow up (6 months), the inclusion of late-stage (KL scale grade 4) knee OA patients, and the lack of a placebo control group. Despite these shortcomings, this study is one of the first prospective randomized trials to compare PRP with MFAT, especially in times in which the increasing accessibility of biologic interventions is outdoing the capability to appropriately study them. We applaud the efforts of the authors and hope to see high-powered, placebo-controlled trials with longer follow-up periods comparing the efficacy of PRP and MFAT, thereby aiding physicians in understanding and selecting appropriate treatment options for their patients suffering from knee OA.

Interestingly, another study was recently published by Zaffagnini et al. (ClinicalTrials.gov identifier: NCT03117608) [15] titled “Microfragmented Adipose Tissue Versus Platelet-Rich Plasma for the Treatment of Knee Osteoarthritis: A Prospective Randomized Controlled Trial at 2-Year Follow-up”, where the authors compared a single injection of MFAT with PRP in terms of clinical outcomes and OA progression. In this study, a total of 118 patients with symptomatic knee OA (KL scale grade 1–4) were randomized 1:1 to either receive a single intra-articular injection (5 mL injection volume) of MFAT or PRP. Patients were examined prior to the injection and at 1, 3, 6, 12, and 24 months utilizing PROM scores, including the International Knee Documentation Committee (IKDC) subjective score, KOOS subscales, EuroQol VAS (EQ-VAS), EQ 5 dimensions (EQ-5D), and VAS for pain. The primary outcomes were the IKDC subjective scores and the KOOS pain subscores at the 6-month follow up. Knees were examined at the baseline and at 6, 12, and 24 months via radiography and high-resolution magnetic resonance imaging (MRI) utilizing the Whole-Organ Magnetic Resonance Imaging Score (WORMS). The PRP utilized was leukocyte-rich (similar to aforementioned Baria et al. study), and the platelets were concentrated to 1000 × 10^3^/µL ± 20% (this concentration was five times higher than the baseline whole blood values). The results demonstrated that both MFAT and PRP led to statistically and clinically significant improvements in all clinical scores except EQ-VAS up to 24 months compared to baseline. No statistical differences in terms of adverse events or failures or primary outcome measures (IKDC subjective score and the KOOS-Pain subscore) were observed between the groups. The radiographic analysis also did not demonstrate any further worsening in OA severity for both treatment groups at all follow-up time points. Similarly, the MRI findings investigated via WORMS also did not show any changes (improvement or signs of disease progression) between both groups at all of the follow-up time points. Interestingly, the subgroup analysis data showed that patients suffering from mild OA did not show any significant differences between the two groups; however, in the case of patients with moderate/severe OA, MFAT showed significantly greater improvement in subjective IKDC scores compared to PRP at 6 months, but this was not observed at the 12- or 24-month follow ups. In summary, similar to the aforementioned study, the results from this trial indicated that the injection of MFAT was not superior to PRP. Similar to the aforementioned study, this trial also has the limitation of the absence of a placebo control group. In spite of this, this study has a high-level study design with a good follow-up duration.

In conclusion, even though they have some constraints, both of these studies presented the scientific community with much required well-executed, prospective clinical trials. These trials, in my view, definitely demonstrated that the administration of MFAT is safe, similar to PRP, and laid the foundation for multi-center, prospective, randomized, placebo-controlled trials to further compare the efficacy of autologous adipose tissue/MFAT with PRP. Additionally, more prospective, randomized, placebo-controlled trials with a larger sample size are warranted to examine the efficacy of MFAT compared to PRP in patients suffering from moderate to severe knee OA to expand on the subgroup analysis findings from Zaffagnini et al.’s trial. As of 26 September 2022, there is only one ongoing clinical trial registered on clinicaltrials.gov (search terms: “knee osteoarthritis” and “PRP” or “Platelet rich plasma” and “fat” or “adipose tissue” or “adipose”). This trial is summarized in Table 1.

## Figures and Tables

**Table 1:** Ongoing clinical trials registered on ClinicalTrials.gov until 28 September 2022, comparing safety and efficacy of autologous adipose tissue with platelet-rich plasma for treatment of knee osteoarthritis.

Study Identifier	Study Phase; Estimated Enrollment (N)	Primary Outcome Measure(s)	Recruitment Status	Country
NCT04321629	Phase II N = 60	Change in The Knee injury and Osteoarthritis Outcome Score (KOOS) (time frame: 1, 3, 6, and 12 months after the treatment): The answers are given (5 Likert boxes), and each question is assigned a score from 0 to 4. A normalized score (100 indicating no symptoms and 0 indicating extreme symptoms) is calculated. Change in the International Knee Documentation Committee 2000 (IKDC 2000) score (time frame: 1, 3, 6, and 12 months after the treatment): The IKDC contains sections on knee symptoms (7 items), function (2 items), and sports activities (2 items). Scores range from 0 points (lowest level of function or highest level of symptoms) to 100 points (highest level of function and lowest level of symptoms). Change in the Western Ontario and McMaster Universities Osteoarthritis Index (WOMAC) score (time frame: 1, 3, 6, and 12 months after the treatment): Due to no Polish version of the WOMAC score being available, it will be calculated from the KOOS score. Higher scores represent worse pain, stiffness, and functional limitations. Change in the quality-of-life score (EQ-5D-5L) (time frame: 1, 3, 6, and 12 months after the treatment): The descriptive system comprises 5 dimensions: mobility, self-care, usual activities, pain/discomfort, and anxiety/depression. Each dimension has 5 levels: no problems, slight problems, moderate problems, severe problems, and extreme problems. The patient is asked to indicate his/her health state by ticking the box next to the most appropriate statement in each of the five dimensions. This decision results in a 1-digit number that expresses the level selected for that dimension. The digits for the five dimensions can be combined into a 5-digit number that describes the patient’s health state. Scores range from 5 points (highest level of function and lowest level of symptoms) to 25 points (lowest level of function or highest level of symptoms). Change in functional status according to The Timed Up and Go Test (TUG) (time frame: 1, 3, 6, and 12 months after the treatment): The result of this test is time-measured with a stopwatch. The time limit for this test is 3 min 30 s. Less time means a better result. Change in functional status according to the 5 Times Sit to Stand Test (5xSTS) (time frame: 1, 3, 6, and 12 months after the treatment): The result of this test is time-measured with a stopwatch. The stopwatch is stopped when the subject sits down after the fifth repetition. Less time means a better result. Change in functional status according to the 10 m Walk Test (10 mWT) (time frame: 1, 3, 6, and 12 months after the treatment): The result of this test is time-measured with stopwatch. The patient is not allowed to run but may use crutches or a walker if needed. Less time means a better result.	Recruiting	Poland
